# Prevalence and risk factors for exposure to *Toxoplasma gondii* in slaughterhouse workers in western Kenya

**DOI:** 10.1186/s12879-021-06658-8

**Published:** 2021-09-11

**Authors:** Elizabeth Anne Jessie Cook, Nduhiu Gitahi, William Anson de Glanville, Lian F. Thomas, Samuel Kariuki, Erastus Kang’ethe, Eric Maurice Fèvre

**Affiliations:** 1 International Livestock Research Institute, Old Naivasha Road, P.O. Box 30709, Nairobi, 00100 Kenya; 2 Institute of Infection Veterinary and Ecological Sciences, University of Liverpool, Leahurst Campus, Neston, CH64 7TE UK; 3 University of Nairobi, P.O. Box 29053, Nairobi, 00625 Kenya; 4 Center for One Health, University of Global Health Equity, Kigali, Rwanda; 5 Kenya Medical Research Institute, P.O. Box 19464, Nairobi, 00200 Kenya

**Keywords:** Slaughterhouse, Abattoir, Kenya, Toxoplasma, Zoonoses, Occupational health

## Abstract

**Background:**

*Toxoplasma gondii* is a zoonotic protozoan parasite infecting warm-blooded animals. Infection in people can occur through ingestion of oocysts passed in the faeces of the definitive hosts; ingestion of bradyzoites in the tissue of infected intermediate hosts; or exposure to tachyzoites in raw milk and eggs. Slaughterhouse workers are considered a high-risk group for *T. gondii* exposure because of their contact with raw meat, although a positive relationship between handling raw meat and *T. gondii* seropositivity has not been demonstrated in all studies. This study aimed to determine the seroprevalence of antibodies to *T. gondii* in slaughterhouse workers in Kenya and identify risk factors associated with seropositivity.

**Methods:**

A survey of slaughterhouse workers was conducted in 142 slaughter facilities in the study area. Information regarding demographics, contact with livestock, meat consumption, and practices in the slaughterhouse was collected using structured questionnaires. Commercial ELISAs were used to detect IgM and IgG antibodies against *T. gondii* and a multi-level logistic regression model was used to identify potential risk factors for seropositivity in slaughterhouse workers.

**Results:**

The apparent prevalence of antibodies to *T. gondii* was 84.0% (95% Confidence Interval (CI) 81.2–86.5%) for IgG and 2.2% (95% CI 1.3–3.5%) for IgM antibodies. All IgM positive individuals were IgG positive. Risk factors for exposure to *T. gondii* were: increasing age (Odds Ratio (OR) 1.03; 95% CI 1.01–1.05); owning poultry (OR 2.00; 95% CI 1.11–3.62); and consuming animal blood (OR 1.92; 95% CI 1.21–3.03).

**Conclusions:**

The seroprevalence of antibodies to *T. gondii* was very high in this population and considerably higher than published values in the general population. Risk factors included age, owning poultry and drinking animal blood which were consistent with previous reports but none were specifically associated with working in the slaughterhouse. In this instance slaughterhouse workers may represent a useful sentinel for the general population where the level of exposure is also likely to be high and may signify an unidentified public health risk to vulnerable groups such as pregnant women. A detailed understanding of the epidemiology of infection is required, which should include an assessment of incidence, mortality, and burden since *T. gondii* infection is likely to have life-long sequelae.

## Background

*Toxoplasma gondii* is a zoonotic protozoan parasite found worldwide. It infects a wide variety of warm-blooded animals including mammals and birds. Sexual reproduction of the parasite occurs in the definitive host (cats) and asexual reproduction occurs in the intermediate hosts (all warm blooded animals) [1]. Infection in people can occur through ingestion of oocysts passed in the faeces of the definitive hosts; ingestion of bradyzoites in the tissue of infected intermediate hosts which is the potential route of transmission to meat handlers including slaughterhouse workers; or exposure to tachyzoites in raw milk and eggs [2]. Vertical transmission is also possible across the placenta [1]. Sporulated oocysts are very robust and can survive in moist soils for more than 12 months [1]. Previously identified risk factors for toxoplasmosis in humans include contact with soil, water or unwashed raw vegetables that might be contaminated with cat faeces, and eating raw meat [2–5].

Toxoplasmosis is generally asymptomatic in immunocompetent individuals but might result in retinitis later in life [6]. Research has also demonstrated a relationship between latent *T. gondii* infection and some mental health disorders [7]. In immunocompromised individuals infection can result in encephalitis with neurological disturbances including seizures and loss of consciousness [8]. Infection in women during early pregnancy can result in foetal death or severe damage to the newborn including, retinochoroiditis, hydrocephalus, seizures and intracerebral calcification [8].

Previously the “gold standard” for definitive diagnosis of *T. gondii* infection was the Sabin Feldman dye test (SFDT), however ELISAs are now used for routine screening in the majority of laboratories [1]. Although the detection of IgG antibodies using ELISA is likely to be later in the course of infection there is relatively good agreement with the SFDT [1, 9, 10]. IgM antibodies may indicate a more recent exposure, but they can persist for several months to years, which means the presence of IgM antibodies does not indicate acute infection nor the time of exposure [11, 12].

There are few reports of exposure to *T. gondii* in Kenya. A study of blood donors in several regions of the country showed the seroprevalence to be 54% [13] and a more recent study of samples collected from women during antenatal clinics in Nairobi, Kisumu and Mombasa reported the prevalence to be 32% [14]. A more targeted study of school children indicated an increase in seroprevalence between preschool and primary school that might be the result of poor sanitation [15]. Recently a study in slaughterhouses demonstrated the prevalence of *T. gondii* by PCR in ruminant slaughterhouse workers to be 34.6% and 100% in chicken slaughterhouse workers [16].

Slaughterhouse workers are considered a high-risk group for *T. gondii* exposure because of their regular contact with raw meat. Although a positive relationship between handling raw meat and *T. gondii* seropositivity has not been demonstrated in all studies [17, 18]. The present study aimed to determine the seroprevalence of antibodies to *T, gondii* in slaughterhouse workers in Kenya and identify risk factors associated with seropositivity.

## Methods

### Study site and population

The study area, in western Kenya on the border with Uganda, is a predominantly rural region characterised by a smallholder mixed crop and livestock system that is broadly representative of the Lake Victoria Basin [19].

A census of all slaughterhouses was attempted with 156 slaughterhouses recruited in the study area – 88 ruminant and 68 porcine. Inclusion criteria were all workers aged over 18 years and present at the slaughterhouse on the day of sampling. Participants were excluded for third trimester pregnancy, severe inebriation, and being aged over 85 years.

### Ethical approval

Ethical approval for this study was granted by the Kenya Medical Research Institute Ethical Review Committee (SCC Protocol 2086). The study was conducted in accordance with the Declaration of Helsinki and each participant gave signed informed consent.

### Data collection and sampling

Data collection was conducted between February and November 2012. Data were collected regarding demographic characteristics of the participants, animal contacts, food consumption practices, and time and role in the slaughterhouse. Questionnaire data were recorded on a Palm operating system (Palm OS) Personal digital assistant (PDA) using Pendragon Forms 5.1 (Pendragon Software Corporation, Libertyville, IL). A clinical officer collected blood from each participant using a 21G or 23G BD Vacutainer® Safetylok™ blood collection set into 10 ml plain BD Vacutainers®.

The locations of slaughterhouses were georeferenced using a handheld GPS device (Garmin eTrex®) and mapped using ArcGIS™ version 10.2.2 (ESRI, Redlands, California, USA).

### Laboratory analysis

Serum samples were screened with the Vir-ELISA Anti-Toxo-IgG (Viro-Immun, Oberursel, Germany) and the Vir-ELISA Anti-Toxo-IgM (Immunocaptureassay; Viro-Immun, Oberusel, Germany) following manufacturer’s instructions. Briefly, the steps for the IgG and IgM ELISAs were: 10 μL of sera were diluted in 1 ml of provided diluent and 100 μL added to each well of the provided precoated plate. For IgG 100 μL of negative control and 4 calibrators were added to the plate and for IgM 100 μL of positive and negative control were added. Plates were incubated at 37 °C for 60 min in a humid environment. Plates were washed four times and 100 μL of peroxidase conjugate added to each well and then incubated at 37 °C for 30 min in a humid environment. Plates were washed four times and then 100 μL of TMB substrate added to each well and incubated at room temperature (21–25 °C) for 15 min. Finally, 100 μL of 0.95 N sulphuric acid was added to each well and the plates read at 450 nm (Synergy HT, Biotek, USA).

### Statistical analysis

Statistical analysis was performed in R (http://CRAN.R-project.org/). The apparent prevalence estimates and the true prevalence estimates were calculated using the *truePrev* function in the *prevalence* package [20] of R accounting for the test sensitivity and specificity reported by the manufacturer of 98.9% and 100.0% for the IgG ELISA and 94.3% and 98.0% for the IgM ELISA respectively. Design-based adjustment was done with the *svydesign* procedure in the *Survey* [21] package in R using sampling weights for each slaughterhouse calculated by dividing the number of expected workers by the number sampled.

Multivariable mixed effects (multi-level) logistic regression models were used to identify risk factors and quantify their association with *T. gondii* seropositivity in slaughterhouse workers. Univariable logistic regression was used to screen variables against *T. gondii* exposure at the individual level. The variables used were those previously reported to be associated with seropositivity to *T. gondii* and included age, gender, contact with livestock, drinking milk, eating meat, role in the slaughterhouse and types of animals slaughtered. All variables are listed in Table 1.

**Table 1:** Results of univariable analysis for risk factors for seropositivity to IgG antibodies to *Toxoplasma gondii* in slaughterhouse workers from western Kenya

Variable	Number (%) n = 737	Number positive (%) n = 619	OR (95%CI)	*p*–value
Individual variables				
Gender				
Female	26 (3.5)	18 (69.2)	1	Ref.
Male	711 (96.5)	601 (84.5)	2.57 (0.98–6.72)	0.055
Age groups				
18–27	167 (22.7)	130 (77.8)	1	Ref.
28–37	227 (30.8)	186 (81.9)	1.42 (0.82–2.46)	0.217
38–47	147 (19.9)	127 (86.4)	1.95 (1.02–3.74)	0.043
48 +	196 (26.6)	176 (89.8)	2.79 (1.46–5.36)	0.002
Age (linear)			1.03 (1.01–1.05)	0.001
Own cattle				
No	252 (34.2)	206 (81.7)		
Yes	485 (65.8)	413 (85.2)	1.34 (0.86–2.10)	0.196
Own sheep				
No	624 (84.7)	524 (84.0)		
Yes	113 (15.3)	95 (84.1)	1.02 (0.56–1.85)	0.947
Own goats				
No	469 (63.6)	391 (83.4)		
Yes	268 (36.4)	228 (85.1)	1.14 (0.73–1.80)	0.558
Own pigs				
No	517 (70.1)	429 (83.0)		
Yes	220 (29.9)	190 (86.4)	1.44 (0.87–2.36)	0.156
Own poultry				
No	104 (14.1)	80 (76.9)		
Yes	633 (85.9)	539 (85.2)	1.97 (1.11–3.48)	0.020
Drink animal blood				
No	339 (46.0)	271 (79.9)		
Yes	398 (54.0)	348 (87.4)	1.96 (1.26–3.06)	0.003
Drink cow’s milk				
No	35 (4.7)	32 (91.4)		
Yes	702 (95.3)	587 (83.6)	0.43 (0.12–1.52)	0.191
Drink goat’s milk				
No	716 (97.2)	601 (83.9)		
Yes	21 (2.8)	18 (85.7)	1.02 (0.27–3.84)	0.980
Eat beef				
No	22 (3.0)	20 (90.9)		
Yes	715 (97.0)	599 (83.8)	0.58 (0.13–2.70)	0.491
Eat pork				
No	230 (31.2)	191 (83.0)		
Yes	507 (68.8)	428 (84.4)	1.18 (0.74–1.89)	0.478
Eat at slaughterhouse				
No	593 (80.5)	494 (83.3)		
Yes	144 (19.5)	125 (86.8)	1.18 (0.64–2.17)	0.600
HIV positive				
No	648 (87.9)	540 (83.3)		
Yes	89 (12.1)	79 (88.8)	1.65 (0.79–3.42)	0.179
Job in slaughterhouse				
Slaughterman/foreman	79 (10.7)	65 (82.3)	1	Ref.
Flayer	579 (78.6)	487 (84.1)	1.13 (0.58–2.18)	0.727
Cleans intestines	43 (5.8)	37 (86.0)	1.09 (0.36–3.32)	0.880
Cleans slaughterhouse	36 (4.9)	30 (83.3)	0.99 (0.32–3.05)	0.989
Other job				
No job	137 (18.6)	115 (83.9)	1	Ref.
Butcher	299 (40.6)	248 (82.9)	0.92 (0.51–1.67)	0.784
Farmer	213 (28.9)	182 (85.4)	1.04 (0.55–1.97)	0.896
Other	88 (11.9)	74 (84.1)	0.96 (0.44–2.12)	0.927
Time as worker (linear)			1.04 (1.01–1.07)	0.019
Slaughterhouse level variables				
Animal type				
Cattle, sheep, goats	274 (37.2)	231 (84.3)	1	Ref.
Cattle only	292 (39.6)	249 (85.3)	1.18 (0.63–2.21)	0.614
Pigs only	171 (23.2)	139 (81.3)	0.83 (0.43–1.60)	0.574

A multivariable mixed effects logistic regression model was developed using variables with a *p*-value < 0.1 in the univariable analysis. The model was developed using the *glmer* function in the *lme4* package [22] with slaughterhouse included as a random effect to account for clustering of the workers. Model selection was conducted using a backwards stepwise approach starting with a full model containing all predictors. Variables with the highest *p*-value were removed in a stepwise fashion until the model with the lowest Akaike information criterion (AIC) was identified. Variance Inflation Factors (VIF) were calculated from the final model to check for collinearity and variables with VIF > 4 were excluded.

## Results

**Fig. 1:**
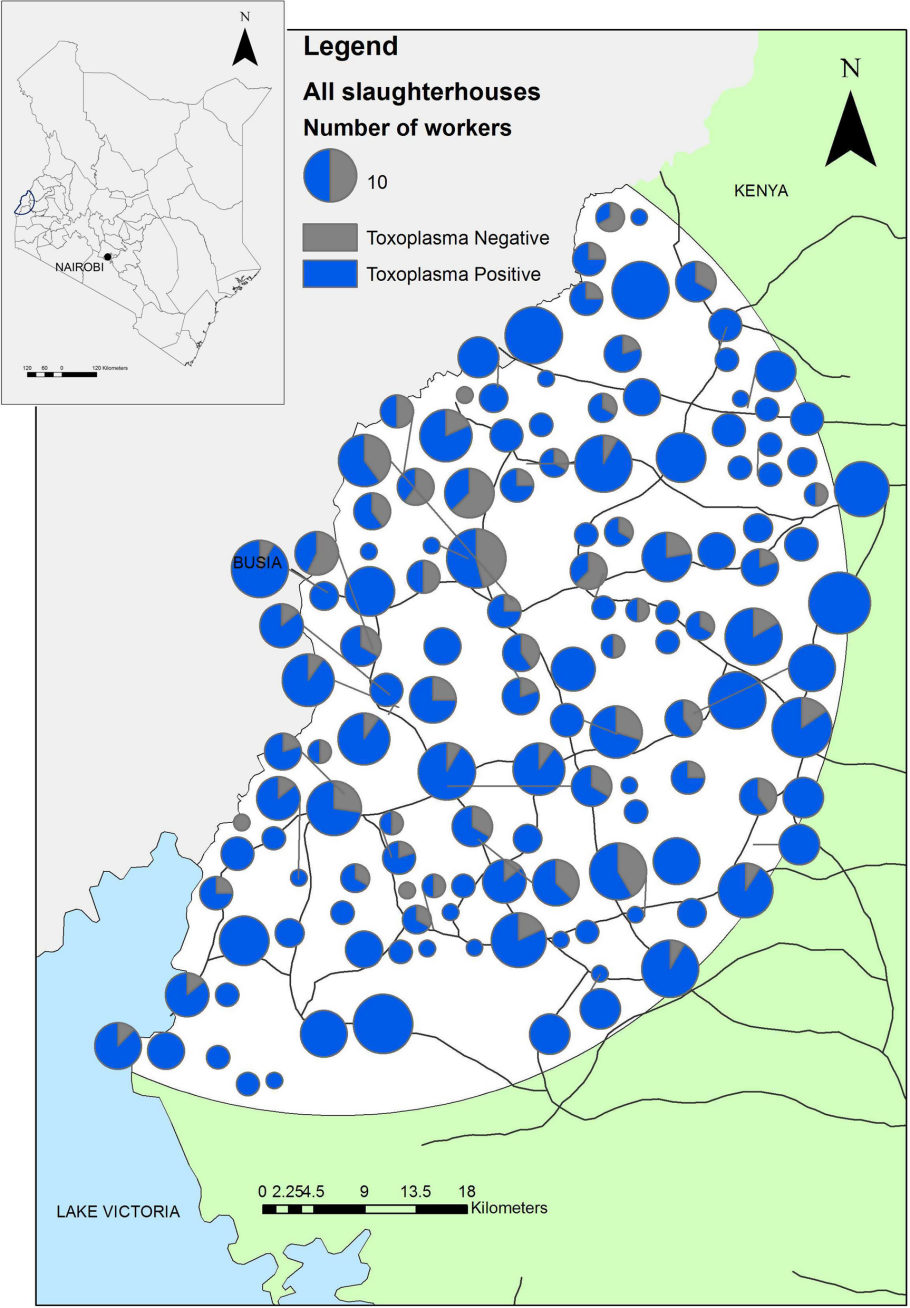
Map of the distribution of *Toxoplasma gondii* seropositive and seronegative slaughterhouses. The size of the charts is proportional to the number of workers in each slaughterhouse. This is an original figure created using ArcGIS

The study recruited 738 slaughterhouse workers from 142 ruminant slaughterhouses. Four ruminant slaughterhouses and 10 pig slaughterhouses refused to participate. Serum samples were available from 737 workers; 619 workers were seropositive for IgG antibodies to *T. gondii* with an apparent prevalence of 84.0% (95% CI 81.2–86.5%). The adjusted prevalence estimate accounting for the study design was 84.3% (95% CI 81.2–87.4%). The true prevalence was 84.8% (95% CI 82.1–87.5%) after adjustment for the sensitivity and specificity of the test. Sixteen (2.2%; 95% CI 1.3–3.5%) workers were positive for IgM antibodies. The adjusted prevalence estimate accounting for the study design was 2.4% (95% CI 1.2–3.7%). The true prevalence was 0.6% (95% CI 0–1.8%) after adjustment for the sensitivity and specificity of the test. All IgM positive individuals were also IgG positive. The distribution of *T. gondii* seropositive workers through the study area is demonstrated in Fig. 1.

The complete univariable analysis for risk factors for *T. gondii* IgG seropositivity in slaughterhouse workers included previously reported exposure variables (Table 1). The variables from the univariable analysis significantly associated with *T. gondii* seropositivity in slaughterhouse workers were: age (OR 1.03, 95% CI 1.01–1.05); owning poultry (OR 1.97, 95% CI 1.11–3.48); drinking animal blood (OR 1.96 95% CI 1.26–3.06); and length of time as a slaughterhouse worker (OR 1.04, (95% CI 1.01–1.07). There was no difference in *T. gondii* seropositivity between slaughterhouse types (Table 1): with seropositivity of 84.2% (230/273) in cattle, sheep and goat slaughterhouses; 85.3% (249/292) in cattle only slaughterhouses; and 81.3% (39/171) in pig only slaughterhouses. The univariable analysis was not conducted for IgM seropositivity as the numbers of positive workers was too small.

The final multivariable model for *T. gondii* IgG seropositivity in individual slaughterhouse workers included the variables gender, age, owning poultry and drinking animal blood. The results are shown in Table 2. Risk factors for exposure to *T. gondii* were: increasing age in years (OR 1.03; 95% CI 1.01–1.05); owning poultry (OR 2.00; 95% CI 1.11–3.62); and consuming animal blood (OR 1.92; 95% CI 1.21–3.03).

**Table 2:** Results of the multivariable analysis for IgG antibodies to *Toxoplasma gondii* in slaughterhouse workers in western Kenya

Variables	OR (95% CI)	p–value	VIF
Individual variables			
Male gender	2.56 (0.94–7.00)	0.066	1.019
Age	1.03 (1.01–1.05)	0.001	1.015
Owning poultry	2.00 (1.11–3.62)	0.022	1.023
Drinking animal blood	1.92 (1.21–3.03)	0.005	1.003

## Discussion

The apparent seroprevalence of IgG antibodies to *T. gondii* in slaughterhouse workers in western Kenya was 84.0% (95% CI 81.2 – 86.5%). This is consistent with reports in slaughterhouse workers in countries from Latin America and Africa, 72% in Brazil [23], 72% in Mexico [24] and 55.8% in Nigeria [25] where the prevalence is reportedly high (> 50%) [1]. This contrasts to Europe where there is low-moderate seroprevalence (10—50%) with reports of *T. gondii* seropositivity in slaughterhouse workers of 45% [26].

We do not have an estimate of the seroprevalence in the general population in this study area to make a direct comparison, however, the prevalence in slaughterhouse workers is higher than that reported in the community (54%) in an earlier study conducted in Kenya [13] and a more recent report from western Kenya of 28.2% [27]. This may indicate that slaughterhouse workers have a higher seroprevalence for antibodies to *T. gondii* but it we cannot rule out that the difference may also be the result of a different test (ELISA versus haemagglutination and microsphere-based immunoassay), different environments, or another occupational exposure.

A previous study in Kenya demonstrated active infections (PCR positivity) in 34.6% of ruminant abattoir workers [16]. In our study it was not possible to determine if infections were active since seropositivity for IgM and IgG does not indicate or distinguish between active and chronic infections [11, 12]. The proportion of seropositive workers increased with increasing age as previously reported [1], however, the highest prevalence may be seen in childhood, particularly in areas with poor hygiene, as demonstrated elsewhere in Kenya [15]. Initial exposure to *T. gondii* occurs in childhood but it is believed that repeated exposure is required to sustain antibody levels [28], which appears to be occurring regularly among slaughterhouse workers in this setting.

There was no relationship between *T. gondii* seropositivity and the role in the slaughterhouse which has previously been identified as a risk factor [23], together with handling meat [23, 29]. A difference in risk between roles in the slaughterhouse might have been expected since the slaughtermen, foremen and cleaners do not handle meat compared to flayers. However, the lack of differential risk between workers might be explained by the method of batch slaughtering conducted in western Kenya where all processes (skinning, evisceration and cutting) are performed in the same spot without a clear demarcation of activities [30]. Workers may also be involved with slaughtering animals at home, with 15% of homesteads in the study area reporting home slaughter which may be an additional route of exposure [19]. Further research is required to compare the prevalence and risk factors in the community to determine if handling or consuming meat is a risk factor for slaughterhouse workers.

In our study seropositivity to *T. gondii* was associated with owning poultry. Poultry are known to be infected with *T. gondii* [31] with the prevalence in backyard chickens reaching 100% in some settings [32]. In western Kenya poultry are free ranging during the day and are kept in the house or kitchen at night [33], and 87.2% of households own poultry [19], which is an intensifying industry in the region [34]. Poultry slaughterhouse workers have been shown to have higher risk of being exposed to *T. gondii* [25, 35]. In this setting poultry are slaughtered at home which may indicate a potential point of exposure for poultry keepers. From the information available it is not possible to determine the potential role of chickens in the epidemiology of *T. gondii* in this setting but these findings suggest they may be important source of infection and further research is required.

Drinking animal blood was a risk factor for *T. gondii* seropositivity in slaughterhouse workers (OR 1.92; 95% CI 1.21–3.03). This is consistent with reports that consuming undercooked animal products is a risk factor for exposure to *T. gondii* [29]. Many workers in this study consume animal blood (54%) and education regarding the preparation of blood before consumption may reduce the risk of exposure.

Infection with *T. gondii* has previously been associated with severe disease in immunocompromised individuals [36]. The prevalence of HIV in the study area is the highest in Kenya [37]. A significant relationship between *T. gondii* infection and HIV was not identified in this study, and the lack of association may indicate a healthy worker effect [38] with affected individuals unable to work.

## Conclusion

The results of this study indicate a high seroprevalence of antibodies to *T. gondii* in a population of slaughterhouse workers in western Kenya, which suggests repeated exposure. None of the risk factors identified here are specifically associated with working in the slaughterhouse. The high infection pressure identified in this study may signify an unidentified public health risk in this region which is a hazard to vulnerable groups such as people living with HIV or pregnant women. There are some gaps in the information which are required to understand the epidemiology of *T. gondii* in this setting such as information regarding raw meat consumption and contact with cats. There is currently no data on the disease incidence or mortality from toxoplasmosis in Kenya. In neighbouring Tanzania a 10 year hospital based study reported the mortality from toxoplasmosis to be 0.08% (188/247,976) of the total deaths recorded [39]. A detailed understanding of the epidemiology of infection is required, which needs to include an assessment of incidence, mortality, and burden since congenital *T. gondii* infection is likely to have life-long sequelae [40]. Future research agendas should consider investing in understanding zoonotic disease risks in sub-Saharan African settings such as this where there is intense animal contact and potential long-term issues.

## Data Availability

The datasets used and/or analysed during the current study are available from the corresponding author on reasonable request.

## Abbreviations

AIC: Akaike information criteria
CI: Confidence interval
ELISA: Enzyme-linked immunosorbent assay
GPS: Global positioning system
Ig: Immunoglobulin
OR: Odds ratio
PCR: Polymerase chain reaction
PDA: Personal digital assistant
SCC: Scientific Steering Committee
SFDT: Sabin Feldman dye test
VIF: Variance Inflation Factors
